# Impact of gillnet soaking time on survival, stress physiology, and muscle quality in Atlantic Cod (*Gadus morhua*)

**DOI:** 10.1017/awf.2025.10023

**Published:** 2025-09-22

**Authors:** Ragnhild Aven Svalheim, Tonje Kristin Jensen, Anette Hustad

**Affiliations:** Nofima AS, Muninbakken 9-13, Breivika, PO Box 6122, N-9291, Tromsø, Norway

**Keywords:** Animal welfare, blood physiology, fillet quality, fishing gear, gillnet, mortality

## Abstract

The aim of this study was to investigate the effects of gillnet soak time to gain a better understanding of fish welfare, mortality, stress, and quality (as measured as muscle haemoglobin) during experimental gillnet fishery of Atlantic cod (*Gadus morhua*). An experimental study was conducted in a large-scale tank at a research facility with 131 wild-caught fish in four groups with gillnet soak times of 0, 2, 12, and 24 h (23–34 fish per soak time). Longer soak time caused higher mortality, with a mortality rate of 0, 7, 18, and 25% in the 0-, 2-, 12- and 24-h groups, respectively. Blood lactate levels were significantly affected by soak time, peaking at 2 h (with the widest confidence interval) and showing their lowest concentrations at 0 and 24 h. Soak time also significantly increased blood glucose and serum cortisol levels. Magnesium, creatinine, and iron increased significantly in all groups compared with control levels, but there was no significant difference between soak times. Haemoglobin content in the loin increased significantly only after 24 h of soak time for live fish. There was no significant increase in haemoglobin in the belly as a function of soak time. However, for all soak times, the belly had significantly more haemoglobin than the loin. Physiological evidence of traumatic injuries and stress were noted prior to increased muscle haemoglobin, meaning that good quality did not necessarily equate to good welfare. However, a higher level of muscle haemoglobin is a strong indication of poor welfare.

## Introduction

There have been a paucity of studies assessing animal welfare in commercial fisheries. Ethical considerations have tended to focus more upon the environmental effects of fisheries and the subsequent implications these may have for the welfare of fish populations (Huntingford *et al.* 2006; Lambooij *et al.* 2012). The concept of animal welfare can be defined in various ways (Korte *et al.* 2007; Broom 2011; Hagen *et al.* 2011; Ohl & Van der Staay 2012), but it has at its core the idea that poor welfare occurs when an animal’s coping capacity has been exceeded, leading, potentially, to chronic stress-related physiology, behavioural anomalies, pathology, and increased mortality.

Within capture fisheries, a wide range of apparatus is used, with selection predicated on target species, geographical region, legislative requirements, and traditional practice. Fishing equipment is deployed in a variety of ways and its impact on fish welfare varies accordingly (Metcalfe 2009). Therefore, any exploration of the effects of the capture process on fish welfare needs to properly take into account these variations in types of gear used. Previous research into animal welfare in fisheries has focused predominantly on trawls and hooks, with only cursory attention paid to purse seines, gillnets, traps, and seines (Veldhuizen *et al.* 2018).

An accurate description of gillnet fishery is provided by He and Pol (2010) who explain that gillnets consist of a vertical mesh panel that is virtually invisible to fish. Once a fish becomes entangled, it is typically caught behind the gill cover and unable to extricate itself. A systematic review by Veldhuizen *et al.* (2018) discovered that there had only been nine articles addressing capture injuries in the context of gillnets. Most of these studies had focused primarily on mortality in terms of fish escaping or being discarded, not to mention capture injuries that impact directly upon the quality of the final product, as opposed to providing an investigation into the actual impact gear has on fish stress, physiology, or overall welfare. There is evidence to suggest that fish caught with gillnets exhibit higher levels of stress compared to alternative methods such as ‘jigging’ (Toledo-Guedes *et al.* 2016). Stressors encountered during capture, such as handling, confinement, and environmental changes, trigger physiological responses that can deplete energy reserves, accelerate rigor mortis, and alter muscle pH, ultimately leading to reduced flesh quality (Lambooij *et al.* 2012; Digre *et al.* 2017). Hook-based capture methods (e.g. longline and jigging) have been associated with better flesh quality compared with gillnet fishing, likely due to differences in stress levels experienced by the fish prior to death (Botta *et al.* 1986; Santos *et al.* 2002; Özyurt *et al.* 2007). Data prepared by the Norwegian Institute of Food, Fisheries, and Aquaculture Research (Nofima) between 2014 and 2020 revealed cod caught with gillnets to show greater occurrence of gear marks, bruising, and discoloured fillets in comparison to those captured using alternative methods such as demersal seines, longlines, and handlines (Joensen *et al.* 2021; Sogn-Grundvåg *et al.* 2022). Nearly 40% of the fish caught by gillnets exhibited significant quality degradation due to inadequate bleeding. Joensen *et al.* (2021) reported soak time (i.e. the duration a gillnet remains in the water to catch fish, starting from when the net is set until it is retrieved) as being the most critical factor influencing the quality of fish caught using gillnets. Additionally, Savina *et al.* (2016) investigated the effect of soak time on catch damage in plaice (*Pleuronectes platessa*) and found that extended soak times were associated with a greater likelihood of catch damage.

Physiological parameters such as blood lactate, glucose, cortisol, and osmolality are commonly used in the objective assessment of stress in fish from wild capture fisheries (Olsen *et al.* 2013; Guida *et al.* 2016; Digre *et al.* 2017; Svalheim *et al.* 2017, 2020; Marçalo *et al.* 2018; Anders *et al.* 2019, 2022). However, wild capture fisheries expose fish to stressors that can trigger a wide range of physiological responses, depending on the species, capture methods, and environmental conditions. These complexities make it useful to consider a broader range of physiological indicators, thereby providing a more comprehensive understanding of fish stress during capture, especially in gillnet fisheries where research is limited.

Cod fisheries in Norway are of great significance commercially, and the gillnet fisheries have been of cultural importance for the coastal communities in Norway for generations, playing a not inconsiderable role in the local economy and the seafood industry as a whole. However, a major challenge facing the industry is the risk of fish dying in the gillnet gear during the soak time as well as injuries incurred by the net leading to muscle blood stains and bruises. Fish that die within the fishing gear are difficult to bleed out properly (Olsen *et al.* 2014) and such is the extent of the muscle discolouration that entire fish may need to be discarded (Ministry of Trade, Industry, and Fisheries 2022). Toledo-Guedes *et al.* (2016) documented a mortality rate of 27.3% for saithe (*Pollachius virens*) captured by gillnets, while 100% of the fish captured by jigging were successfully hauled on board alive. Similarly, Santos *et al.* (2002) reported that discards constituted 42% of the gillnet’s total catch for European hake *(Merluccius merluccius)*, but only 7% for longlines targeting the same species. These increased mortality rates from gillnet fisheries are strongly suggestive of gillnets not being optimal in terms of welfare. Despite this, to the best of our knowledge, no link has been made between the stress and welfare of fish caught by gillnets and the level of quality of the overall product, nor has there been any scientific documentation regarding how different soak times affect survival, stress, and quality in Atlantic cod (*Gadus morhua*).

This study aimed to investigate the physiological stress and welfare implications associated with the gillnet capture of Atlantic cod. Specifically, we examined how different gillnet soak times may affect survival, stress levels, and quality of cod in terms of haemoglobin in the muscle. It was our hypothesis that longer soak times would lead to higher mortality, stress levels and haemoglobin in muscle thus impinging on the welfare and quality of the fish. Through extending beyond the more commonly explored physiological parameters we sought to garner a better understanding of the welfare implications of gillnet capture in Atlantic cod.

## Materials and methods

### Study animals and husbandry

A total of 200 wild Atlantic cod with a mean (± SD) weight of 1.2 (± 0.8) kg (see Table 1 for detailed biological data) were captured using commercial cod pots in Malangen outside of Tromsø, Norway (69°36’40.9” N–18°54’48.6” E at 74.43 m depth, and 69°38’53.3” N–18°53’13.8” E at 71.32 m depth) on the 29^th^ of September 2021. Of these, 131 were used in the experiment. Immediately following capture, fish were placed into an onboard holding tank supplied with running seawater and transported to the Aquaculture Research Station in Tromsø, Norway. At the research station, the fish were held indoors in a 5-m diameter tank with a volume of 50,000 L under a natural photoperiod (transparent roof, 69°N) for 16 days prior to the onset of the experiment. The tank was supplied with ambient sand-filtered seawater (200 L per min) pumped from the sea outside the aquaculture station. At the start of the experiment, the temperature of the seawater was 9°C, but it decreased to 5°C during the live holding and the experiment. Oxygen levels in the tank were measured once a week (YSI Pro ODO, YSI Incorporated, Xylem Inc, Yellow Springs, OH, USA), and all oxygen values during live holding were above 83.6% saturation. Due to the duration of the experiment, starving the fish was not deemed appropriate from an animal welfare perspective and thawed Atlantic herring (*Clupea harengus*) was provided as feed. Additionally, prolonging the experiment to accommodate a fasting period was not feasible given constraints on how long fish could be kept at the station.

**Table 1.** Mean (± SD) soak time, fish characteristics, and physiological indices for Atlantic cod (*Gadus morhua*) in a tank-based gillnet study (n = 131)

Soak time (h)	N	Weight (g)	Length (cm)	K	HSI	GSI
Control	23	1,890 (± 1,266)	55.7 (± 13.8)	0.95 (± 0.11)	0.03 (± 0.01)	0.02 (± 0.01)
0	23	1,134 (± 669)	47.8 (± 8.4)	0.94 (± 0.09)	0.02 (± 0.01)	0.02 (± 0.01)
2	27	980 (± 443)	55.4 (± 33.1)	0.91 (± 0.19)	0.03 (± 0.01)	0.02 (± 0.01)
12	34	1,006 (± 320)	46.8 (± 4.6)	0.96 (± 0.13)	0.03 (± 0.02)	0.01 (± 0.01)
24	24	932 (± 331)	46.5 (± 5.0)	0.90 (± 0.12)	0.03 (± 0.02)	0.02 (± 0.01)

K: Fulton’s condition factor; HIS: hepatosomatic index; GSI: gonadosomatic index. Note that soak time is defined here as the duration from when the fish were caught in the gillnet until retrieval.

### Ethics statement

All experimental protocols were authorised by the Norwegian Animal Welfare Authority (Mattilsynet, FOTS licence ID: 23410) with the experiment designed in accordance with the 3Rs. There was no alternative to the use of live animals. Power analysis estimated optimal sample sizes and all fish were euthanased using a percussive blow to the head prior to any invasive sampling beyond gillnet capture.

### Experimental set-up

The experiment was conducted in three replicates over 13 days from October to November 2021. Four soak times of 0, 2, 12, and 24 h were used. Here, soak time is defined as the time from when the fish were caught in the gillnet until they were retrieved. Control fish were randomly sampled from the tank using a hand net at three different time-points per replica prior to the netting event.

A large gillnet consisting of two separate nets with mesh sizes 45 and 60 mm attached to one 720 × 260 cm (length × height) gillnet was run across the diameter of the holding tank. After 7–13 min, the desired number (minimum seven fish per replica for each soak time) of fish were caught in the gillnet. In most cases, the fish swam into the gillnet of their own volition. However, as the experiment progressed and fish density in the tank decreased, a hand net was used to gently cajole fish into the gillnet. The gillnet was then gently hauled and placed in a dry fish bin before being immediately relocated to a smaller tank (4,000 L) which contained identical water conditions to the previous tank. However, the gillnet was not deployed in the same manner as a result of the tank’s reduced diameter. Fish were exposed to the air for 5–10 s, and the ambient temperature was 10.0°C and both these parameters remained consistent across all soak time treatments. The fish were kept in the gillnet for 0, 2, 12 and 24 h prior to sampling.

### Sampling procedure

Fish were lifted individually from the water, and their state was assessed based on physical responses to handling. Consciousness was evaluated using the vestibular-ocular reflex (VOR), which was observed by checking for eye movement in response to head rotation while the fish was in the net. Additionally, fish were monitored for opercular movements and responsiveness to handling. The absence of VOR, opercular movement, and reaction to stimuli has been established as a reliable method for confirming death rather than transient unconsciousness (Raby *et al.* 2011). Given the complete absence of these indicators it was possible to determine death had taken place with a high degree of confidence. However, we acknowledge a small possibility of recovery under certain conditions. For the purposes of this study, fish meeting these criteria were classified as dead.

**Figure 1.**
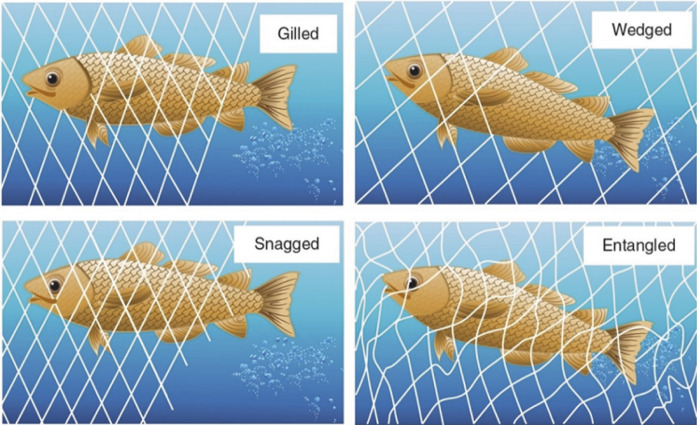
The four modes of gillnet capture: gilling, wedging, snagging, and entangling observed for Atlantic cod (Gadus morhua) in a tank-based gillnet study (n = 131) (modified from He & Pol 2010).

Capture mode was categorised into four types: entangling, wedging, gilling, and snagging. Entangling refers to fish becoming wrapped or twisted in the net by various parts of their body, wedging occurs when a fish is held tightly by the mesh around a specific part of its body, usually just behind the gill covers, gilling occurs when the fish is held by the mesh around the gill covers, and snagging describes fish caught by other body parts such as fins or spines (Figure 1). These definitions were used consistently throughout the study to classify how fish were retained in the gillnet. Capture mode was recorded only for fish found dead in the net since escaped fish could not be reliably assessed. Analyses, therefore, did not include fish that were snagged but subsequently escaped.

Living fish were euthanased by a percussive blow to the head and individually tagged with a Floy t-bar anchor tag in the operculum. Then, blood was collected from the caudal vessels using a 3-ml clot activator serum vacutainer (Vacutest Kima, Arzengrande, Italy) with 0.9 × 38 mm needles (BD Diagnostics, Franklin Lakes, NJ, USA). Concentrations of lactate and glucose were obtained from samples of whole blood, using the point-of-care device LactatePro™2 (Arkray Europe BV, The Netherlands) and Accu-Chek (Roche Diabetes Care, GmbH, Germany), respectively (Brown *et al.* 2008). Blood samples were centrifuged (3000× g, 10 min, 4°C), and serum was frozen at –80°C before analyses. Serum cortisol was quantified using a solid-phase Enzyme-linked Immunosorbent Assay (ELISA) Kit (Demeditec Diagnostics GmbH, Kiel, Germany) following the manufacturer’s instructions. Remaining serum parameters were measured using an automated Pentra C400 (Horiba Medical, Kyoto, Japan). Fish that died in the gillnet were individually tagged and followed the same procedures as the living fish, except for blood sampling, which was only performed on live fish.

Immediately following blood collection, fish were removed from the gillnet, bled by cutting the isthmus and exsanguinated in running water for 30 min in a 200 L tank (–6.9 to 8.3°C). This procedure closely resembles what commonly occurs in commercial gillnet fisheries. Next, weight (g), length (cm) and sex of each fish were registered. The liver and gonads were also weighed (g) to determine hepatosomatic (HSI) and gonadosomatic indices (GSI) by tissue weight × 100/total weight. The fish were then gutted, cleaned in running water and placed on ice in 70-L fish crates with the belly cavity facing downward for 72 h to ensure post-rigor filleting. Fish were then manually filleted with the skin retained and the black peritoneum was removed. Next, the fillets were cleaned in running freshwater, vacuum-packed, and stored at –20°C for four weeks before thawing and analysis. Chemical analysis of haemoglobin was conducted using left fillets. Both the fish that died in the net and those that survived were used for haemoglobin measurements. Fish and fillets were handled gently throughout processing to minimise any impact on quality.

### Chemical analysis of haemoglobin

Prior to chemical haemoglobin analyses, the sealed, vacuum-packed fillets were thawed in a clean container with 50 L of chilled water (1°C) for 1 h. Next, the fillets were taken out of the vacuum bags, skinned, and the loin and belly were cut and minced in a food processor (Bosch MultiTalent 3 MCM3110W, BSH Hausgeräte GmbH, Münich, Germany). Total haem protein was determined as described by Chaijan and Undeland (2015) by weighing approximately 2 g of minced fish sample (loin and belly) in a 50-ml centrifuge tube (Falcon Blue MacTM, Polypropylene Conial Tube, Becton Dickinson Labware, NJ, USA) and adding 6 ml 0.1 M phosphate buffer, pH 7.0, containing 5% SDS. The samples were homogenised using an Ultra Turrax T25 homogeniser (IKA Werke GmbH, Staufen, Germany) for 30 s at 6,000 rpm. The homogenate was subjected to 85°C for 1 h in a temperature-controlled water-bath (Julabo SW22, JULABO Labortechnik GmbH, Germany). After cooling for 10 min at room temperature (25°C), the solution was centrifuged at 5000×g for 15 min (Eppendorf®, Germany). The absorbance of the supernatant was read at 535 nm using a Visible Spectrophotometer Genesys 20 (Thermo Scientific^TM^, Waltham, MA, USA) and compared with a standard curve based on bovine haemoglobin with concentrations ranging between 0 and 50 μM.

### Statistical anlysis

We tested for significant differences among treatment groups to assess whether weight, length, hepatosomatic index (HSI), and K-index differences could confound the biomarker analyses. Given that normality assumptions were violated for weight, HSI, and K-index, we employed Welch’s ANOVA, which accounts for unequal variances and is more robust to deviations from normality. We used a standard one-way ANOVA for length, which met normality assumptions.

The analyses revealed no significant weight, length, HSI, or K-index differences across treatment groups (*P* > 0.05). We determined that adjusting for these variables in subsequent biomarker analyses was unnecessary, as there was no evidence of systematic variation that could bias physiological responses.

To assess the effect of soak time on survival, we initially attempted a generalised linear mixed model (GLMM) with a binomial distribution and logit-link function, including soak time as a fixed effect and replication as a random effect. However, this model failed to converge, likely due to the dataset’s low mortality rates and separation issues.

Given the low number of deaths in some groups, we applied non-parametric bootstrapping with 1,000 resamples to estimate the effect of soak time on survival. Bootstrapping allows for a more robust estimation of survival differences while avoiding issues related to complete separation in logistic regression. The model estimated bootstrapped regression coefficients (log-odds of survival) and bias-corrected 95% confidence intervals (CIs) for each soak time group.

To determine the effects of soak time on physiological biomarkers in fish, we employed Generalised Linear Mixed Models (GLMMs), considering replicate as a random effect. Given the biological nature of the response variables, continuous measures of blood chemistry that are strictly non-negative and often right-skewed, we first evaluated the most appropriate distribution for modelling the data. To achieve this, we compared Gaussian, Gamma, and inverse Gaussian distributions, using Akaike Information Criterion (AIC) values to assess model fit. The Gamma distribution consistently yielded the lowest AIC values and was therefore chosen for the model.

Furthermore, link function selection was refined by testing the identity, log, and inverse links, with the final choice determined based on AIC values and residual diagnostics. After confirming the appropriate distributional assumptions, we assessed Intraclass Correlation Coefficients (ICCs) to quantify the proportion of variance explained by the random effect (replicate). Several biomarkers, including cortisol and creatinine, exhibited exceptionally high ICC values (≥ 0.96), indicating that nearly all variability was attributed to differences between replicates. This suggested that replicate was not a purely random factor but systematically influenced the response, warranting its inclusion as a fixed effect in a Generalized Linear Model (GLM) instead.

For biomarkers where ICC values were negligible (≤ 0.001), such as lactate, haemoglobin, iron, and magnesium, the replicate was removed from the model, as it contributed minimal variability. Conversely, for glucose and urea, which had moderate ICC values, the replicate was retained as a random effect within a GLMM framework, ensuring an appropriate balance between model complexity and variance partitioning.

Model selection was conducted by comparing AIC and Bayesian Information Criterion (BIC) values across different model structures. This rigorous approach ensured that the most parsimonious and biologically appropriate model was chosen for each biomarker. All statistical analyses were conducted in R (v4.2.3; R Studio Team 2022) using the lme4 and car packages. The final statistical framework allowed for a robust assessment of the effects of soak time on physiological stress and metabolic responses, while accounting for replicate-level variation, ensuring reproducible and biologically meaningful interpretations of the results.

## Results

All rounds of soak time treatments resulted in fish getting caught in the gillnet. It took a mean (± SD) of 10 (± 3) min to capture 9 (± 3) fish in each treatment replicate. Immediately after being caught in the net, all fish exhibited increased swimming activity in an attempt to escape, including rapid bursts and strong tail beats. We also observed firm tail flaps and lateral flexions, creating a rotation that, in most cases, caused the net to entangle more around their heads and bodies. The physical activity occurred primarily in the first 1–2 min following capture; after this, the fish would make a few rapid jerks whenever a new fish got caught. The fish already in the net would only jerk a few times, but did not undergo the same intense struggle as newly caught fish.

**Figure 2.**
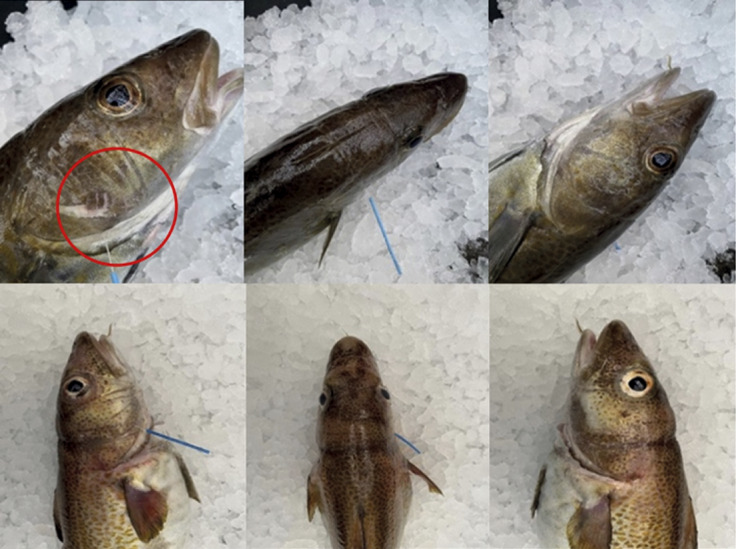
Example of gear marks in Atlantic cod (*Gadus morhua*) after 2 and 24 h of soak time in a tank-based gillnet study (n = 131). The figures show two cod that died in the gillnet after (top) 2 h and (bottom) 24 h soak time. The gear marks show that the gillnet was wrapped around the operculum. The red circle indicates visible skin abrasion caused by the gillnet.

A total of five fish were caught by snagging, but these would escape the net during/after transfer to a second tank and were excluded from the experiment. All fish that died in this experiment were caught by entangling and gilling (Figure 2). Bootstrapped confidence intervals showed that fish exposed for 12 and 24 h had significantly lower survival compared with the control group (12 h: 95% CI [–19.37, –16.89]; 24 h: 95% CI [–19.81, –17.37]). Exposure for 2 h resulted in reduced survival, but the effect was not statistically significant (95% CI [–18.23, 0.00]). There were some variations between the replicates (see Table 2 for details).

**Table 2.** Number of alive and dead Atlantic cod (*Gadus morhua*) at different soak times (0, 2, 12 and 24 h) across multiple replicates in a tank-based gillnet study (n = 131)

Replicate	Control		0 h		2 h		12 h		24 h	
	Alive	Dead	Alive	Dead	Alive	Dead	Alive	Dead	Alive	Dead
1	9	0	7	0	11	0	10	2	9	1
2	7	0	10	0	11	1	11	3	5	4
3	7	0	6	0	3	1	7	1	4	1
Total	23	0	23	0	25	2	28	6	18	6
%	100	0	100	0	93	7	82	18	75	25

NB: Each row represents a separate replicate, and the total number of fish per group is summarised at the bottom. Percentages indicate the proportion of live and dead fish within each group. The term ‘replicate’ refers to individual experimental runs with repeated measurements under the same conditions.

**Figure 3.**
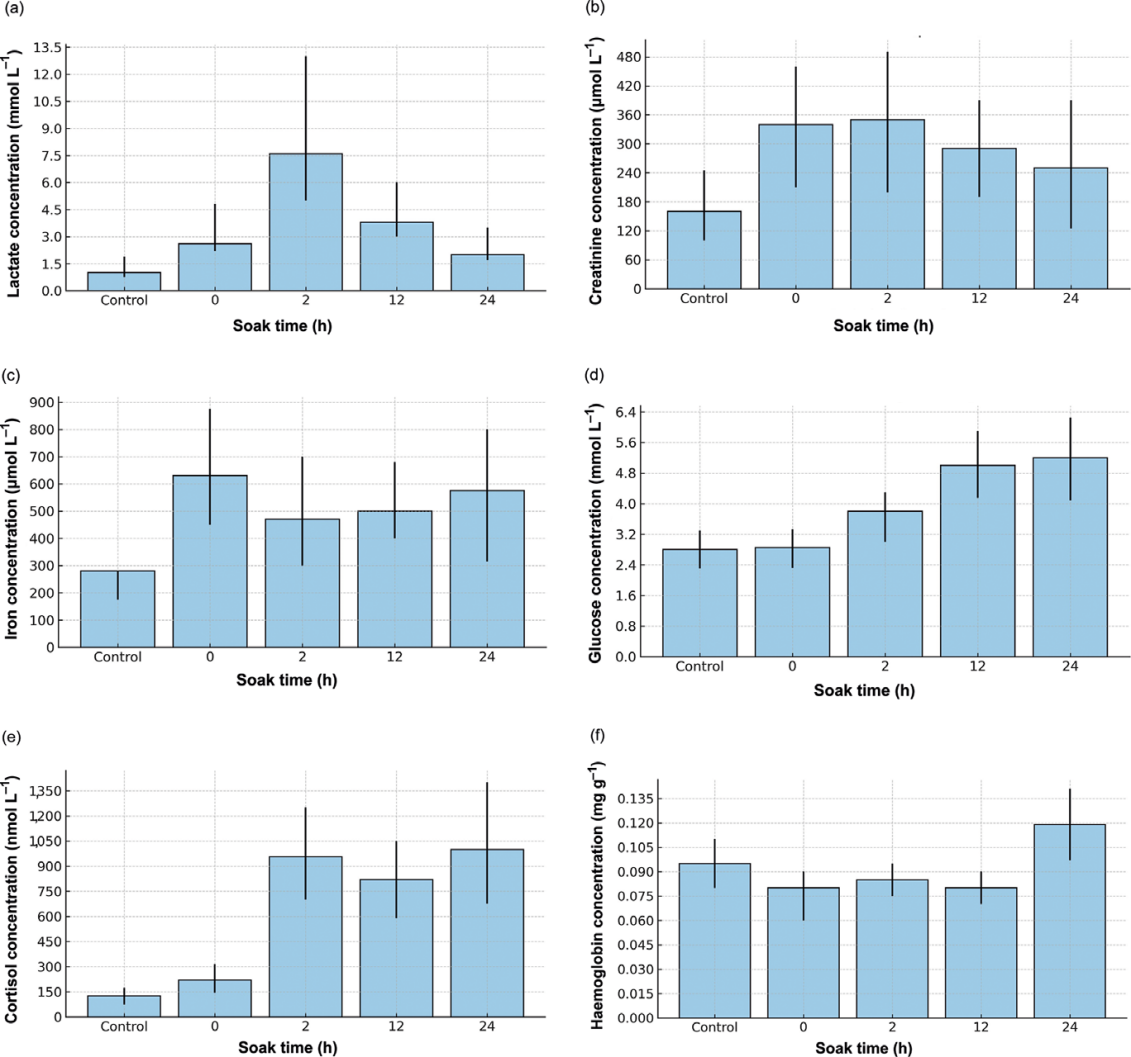
Physiological parameters of Atlantic cod (*Gadus morhua*) measured at different soak times (0, 2, 12 and 24 h) following gillnet capture in a tank-based study (n = 131). Parameters measured were (a) lactate (mmol L^–1^), (b) creatinine (μmol L^–1^), (c) iron (μmol L^–1^), (d) glucose (mmol L^–1^), (e) cortisol (nmol L^–1^) and (f) haemoglobin in the loin muscle (mg g^–1^ muscle). Each bar represents the mean of three independent replicates, with 95% confidence intervals shown. Statistical analyses were carried out using GLM or GLMM models.

Lactate levels differed significantly difference groups (*P* < 0.001) (Table 3), with significant differences between the control and 0-, 2-, and 12-h groups, as well as between 2 and 24 h (Figure 3a). However, lactate levels at 24 h did not differ significantly from the control. Similarly, glucose levels were significantly different between groups (*P* < 0.001) (Table 3), with *post hoc* analysis indicating significant differences between the control and 12-h groups, the control and 24-h groups, as well as between 12 and 2 h (Figure 3d). Glucose levels were higher in exposed fish compared with the control at multiple time-points.

**Table 3.** Results of statistical analyses of physiological parameters for Atlantic cod (*Gadus morhua*) in a tank-based gillnet study (n = 131)

Variable	*P*-value	Model	*P*-value replicate	Distribution	Link function
Glucose (mmol L^–1^)	< 0.001	GLMM (Replicate, ICC = 0.036)		Gamma	identity
Lactate (mmol L^–1^)	< 0.001	GLM (Replicate, ICC = 0.000)		Gamma	inverse
Haemoglobin in loin muscle (mg g^–1^)	0.008	GLM (Replicate, ICC = 0.000)		Gamma	identity
Haemoglobin in belly muscle (mg g^–1^)	0.065	GLM (Replicate, ICC = 0.001)		Gamma	identity
Cortisol (nmol L^–1^)	< 0.001	GLM (Replicate, ICC = 1.000)	< 0.001	Gamma	identity
Iron (μmol L^–1^)	0.005	GLM (Replicate, ICC = 0.000)		Gamma	identity
Urea (mmol L^–1^)	0.422	GLM (Replicate, ICC = 0.113)	0.0171	Gamma	identity
Magnesium (mmol L^–1^)	< 0.001	GLM (Replicate, ICC = 0.000)		Gamma	identity
Creatinine (μmol L^–1^)	< 0.001	GLM (Replicate, ICC = 0.960)	*P* = 0.091	Gamma	identity

Cortisol levels exhibited a highly significant difference between groups (*P* < 0.001) (Table 3), with *post hoc* analysis revealing significant differences between the control and all time-points (0, 2, 12, and 24 h), as well as between 0 and 2 h (Figure 3e). Cortisol levels at 24 h remained significantly different from the control.

Haemoglobin levels in the loin showed a significant difference (*P* = 0.0079) (Table 3), with *post hoc* analysis revealing significant increases between 0 and 24 h and between 12 and 24 h (Figure 3f). However, haemoglobin levels in the belly flap did not reach statistical significance (*P* = 0.065).

Magnesium levels showed a statistically significant difference (*P* < 0.001) (Table 3), with significantly higher concentrations at 12 and 24 h compared with the control. Iron levels were also differed significantly between groups (*P* = 0.0048) (Figure 3c), with *post hoc* analysis indicating a significant increase at 0 h compared with the control. Creatinine levels were statistically significant (*P* = 0.0004), but *post hoc* tests did not detect any pair-wise significant differences (*P* > 0.05), suggesting that while an overall effect was present, individual group comparisons did not reach significance (Figure 3b).

Urea levels did not show a significant overall difference (*P* = 0.4223) (Table 3), though replicate variation (*P* = 0.0171) may have influenced the results.

## Discussion

We investigated how different soak times in gillnets affected mortality, physiological stress, and muscle haemoglobin of Atlantic cod. Our findings indicate that extended soak periods result in higher mortality rates, increased physiological stress, and a potential reduction in meat quality due to alterations in muscle haemoglobin. Whilst our results would suggest that shorter soak times may improve fish welfare, we recognise that there are a host of operational and economic factors that must be taken into account when determining soak times in commercial fishing operations. For instance, it may be neither practical nor cost-effective for fishermen to use very short soak times, since catch rates and associated costs are key factors to consider. Our study was focused purely on the benefits of reducing soak times as they related to fish welfare. However, more discussion is needed on how such practices can be realistically implemented, given current management constraints and financial limitations.

Lactate levels increased significantly after 2 h of soak time, and of the measured time-points, the levels were highest after 2 h and significantly lower at 24 h. Lactate is commonly used as an indicator of oxidative stress in fish (Moon 2011; Sopinka *et al.* 2016), and the increase observed here illustrates that the escape response during capture is sufficient for anaerobic metabolism to occur leading to the production of lactate in the white muscle. However, it is also possible that functional hypoxia, unrelated to exercise, contributed to the increased lactate. Also, the gilling process could have impaired respiration, leading to insufficient oxygen supply and a subsequent shift to anaerobic metabolism (Clow *et al.* 2017). The peak after 2 h is in accordance with other studies of oxidative stress (Milligan 1996; Olsen *et al.* 2008; Svalheim *et al.* 2017, 2020) and fits with our observations that the escape response was most pronounced directly following capture and that the fish showed low activity for the rest of the soak time. We only sampled blood from fish that were still alive. The reduction of lactate after 12 to 24 h indicates that these fish could ventilate, due to the need for oxygen for the resynthesis of lactate into glycogen. However, the large confidence interval after 2 h may indicate that some of these fish struggled to acquire enough oxygen, while others may have either: (i) successfully ventilated and begun to recover from the anoxic stress; or (ii) expressed a lower intensity escape response at the initial capture.

Two hours following exposure, cortisol levels showed a significant increase, suggesting acute stress and levels remained elevated throughout the 24-h replicate, indicating a persistent stress reaction. This extended rise in cortisol can impact fish physiology in various ways, including metabolic alterations. Other studies on Atlantic cod have shown recovery to be possible with cortisol levels decreasing significantly and even returning to basal levels 24 h following acute stress (King & Berlinsky 2006; Olsen *et al.* 2008; Svalheim *et al.* 2017, 2020). Since no such returns were observed in this study, it strongly suggests that being caught in a gillnet is a continuous stressor until stunning or slaughter. Glucose levels also increased significantly, often due to cortisol-mediated gluconeogenesis (Milligan 1996). The modest increase in glucose suggests that while metabolic adjustments were occurring, they were less pronounced than the cortisol response. This could be due to various factors, including the fish’s ability to mobilise other energy reserves or the initial levels of glucose in the blood. The interaction between cortisol and glucose is important for understanding the stress physiology of the fish, as the increase in blood glucose availability from cortisol stimulation of gluconeogenesis provides energy for the fight-or-flight response. In the present study, this metabolic response is probably meant to aid the fish in their attempt to flee and endure the stress of entanglement (Hemre *et al.* 1991; King *et al.* 2006; Olsen *et al.* 2008). However, the relatively low glucose response suggests that this mechanism might be secondary or complementary to other, more dominant stress responses, such as lactate or magnesium.

Stress can also cause a shift from intracellular to extracellular magnesium, which acts to diminish the stress response mediated by catecholamines (i.e. adrenaline) and glucocorticoids (i.e. cortisol) (Cuciureanu & Vink 2011) and positively affects the affinity of haemoglobin for oxygen (Flatman 2003; Wells 2009). We found an immediate increase in serum levels of magnesium. However, it is unclear if this is primarily a result of the shift from intracellular to extracellular magnesium or, for example, dehydration and osmotic imbalance. Olsen *et al.* (2008) found that intestinal permeability increases following an acute stressor, and as most of the magnesium in saltwater fishes is excreted via the faeces, it is possible that the fish were experiencing osmotic water loss and excess ion gains in addition. Magnesium is involved in a wide variety of cellular processes, including anaerobic and aerobic metabolism, bioenergetic reactions, regulation of metabolic pathways, signal transduction, ion channel activity, cell proliferation, differentiation, apoptosis, angiogenesis, and membrane stabilisation (Nishizawa *et al.* 2007; Wolf *et al.* 2007; Szewczyk *et al.* 2008). However, the exact cause of the increase is difficult to determine. Nevertheless, the significant increase in magnesium levels immediately following entanglement suggests that magnesium responds very quickly to the stress of capture by gillnet. This indicates that magnesium could be an early indicator of acute stress in fish and a valuable biomarker for detecting and assessing short-term stress responses.

Serum creatinine and iron increased immediately after capture and remained elevated for the longer soaks. This is in line with previous studies that have shown a decrease in the expression of muscle hepcidin, a key regulator of iron metabolism, allowing more iron to be available for metabolic needs (Shen *et al.* 2019). Furthermore, the increased serum iron may indicate that gillnets obstruct the fish’s ability to fully ventilate, as this decrease in hepcidin can indicate hypoxia (Subramaniam & Wallace 2014). It is worth noting that while the changes in iron levels were statistically significant, the magnitude of these changes is relatively small. However, from a welfare perspective, even small changes are meaningful and should not be overlooked, as they contribute to a cumulative stress response.

Creatinine is produced in muscle and serves as an energy buffer that helps stabilise and maintain cellular energy balance. While prolonged elevation of creatinine can indicate kidney damage as creatinine is excreted via glomerular filtration (Amin & Hashem 2012), the observed increase in creatinine in the present study is more likely due to acute stress rather than kidney dysfunction. A reduced glomerular filtration rate could explain increased creatinine, but we did not observe a corresponding rise in urea concentration, which would be expected during severe kidney failure. Additionally, while creatinine is often linked to muscle damage in certain conditions (Threlfall *et al.* 1984; Iapichino *et al.* 1985), the rapid increase in creatinine observed here is inconsistent with muscle injury. The increase occurred immediately after the fish were captured in the net, suggesting that it is more likely related to the activation of stress pathways rather than muscle damage. This could be due to oxidative stress induced by the physical exertion of escape attempts and the potential restriction of opercular function. Other studies have shown that conditions leading to oxidative stress also disrupt creatinine metabolism, rapidly increasing creatinine levels (Mohammed et al. 2015). However, the rapid increase of creatinine in our study does not tie in with the assumption of muscle damage, as the increase occurred directly after getting caught in the net. Therefore, it is more likely that the increase is due to oxidative stress from activity and potential opercular restrictions. The fish in our study showed a two-fold increase in creatinine after 24 h in the gillnet compared with control levels. Other studies have shown that rainbow trout (*Oncorhynchus mykiss*) regain basal levels of creatine 24 h post-stressor when allowed to recuperate (Milligan 1996). This supports our hypothesis that being caught in a gillnet is a continuous stressor until stunning or slaughter.

Longer soak time was associated with increased mortality, higher stress levels and a reduction in meat quality as measured by the amount of muscle haemoglobin. Our experimental study showed mortality in cod to be between 18 and 25% after 12 to 24 h, respectively. Although Toledo-Guedes *et al.* (2016) did not compare different gillnet soak times, they found a mortality of 27.3% in saithe caught by gillnets soaked between 12 and 17 h, which aligns with our study. However, the fish we used in the present study were considerably smaller than those that tend to be caught by commercial vessels operating with gillnets (gillnet mesh size of 45 and 60 mm in the present study versus minimum mesh size of 156 mm in the commercial fishery for cod north of 62^0^) (Ministry of Trade, Industry and Fisheries 2022). Smaller fish may have different physiological responses to gillnet capture than larger fish because these fish are generally more vulnerable to capture-related stress and injury, which could result in higher mortality rates (Cook *et al.* 2018; Veldhuizen *et al.* 2018). As such, the results observed in this study, which used smaller fish, may not directly reflect the outcomes in the commercial fishery where larger fish are typically caught. Future studies should investigate whether the observed differences in mortality and physiological stress are consistent across different sizes of fish and how these findings relate to larger fish caught in commercial fisheries.

In commercial fisheries, there will always be uncertainty regarding how long the fish has been in the net, as it can legally be anywhere from 0 to 24 h. Therefore, a significant advantage of our experimental set-ups was that we were able to control the environment and the timing of fish entering the fishing gear. Svalheim *et al.* (2020) also demonstrated the difficulties of comparing experimental set-ups with commercial capture. In a study simulating trawl fisheries with extreme crowding, Svalheim *et al.* (2020) found 18% mortality after 3 h of crowding and 91% after 5 h of crowding. Olsen *et al.* (2013) found 27% mortality with 5 h of hauling time. In addition, the allostatic load may be greater in commercial fishing, where the fish are exposed to greater changes in depth, current conditions, temperature, predation, sounds, etc.

However, the findings from the trawl studies, whether experimental or commercial, still presented high mortality at lower exposure times (i.e. time in gear). In contrast, we found 7% mortality after 2 h of soak time of gillnets and 25% after 24 h. This might indicate that gillnets are less stressful per time unit compared with densely packed trawls or seines. This does not necessarily equate to gillnets representing gentler fishing gear, since the total fishing time is usually much longer for gillnets (legally up to 24 h in Norway) (Ministry of Trade, Industry and Fisheries 2022) compared to trawl hauls (30 min–5 h). The time duration of the potential stress needs to be taken into account during any discussions on benign fishing gear or otherwise.

Fish that died in the gillnet had the net wrapped around the operculum, probably making it difficult for the fish to ventilate, ultimately leading to death by asphyxiation. Fish survival was highest (93%) for those only caught in the gillnet for 2 h, compared with 12 (82%) and 24 h (75%), but even when soak time was short (i.e. 2 h), dead fish were observed with the gillnet wrapped around the operculum. Fish caught in the net by snagging or wedging (He & Pol 2010) could move the operculum, allowing them to survive the capture process. Clearly, how fish are caught in the gillnet (i.e. gilling, wedging, snagging, or entangling) affects the survival of the catch. This may have further consequences for catch quality because fish that die in the fishing gear are difficult to bleed out properly, leading to red muscle discolouration (Olsen *et al.* 2014). Although gear modifications could theoretically reduce the impact of gilling, exploring such solutions would require further research into their technical and economic feasibility, not to mention their acceptance by commercial fishers. These aspects lie beyond the scope of the current study but should be considered in future research.

Concerning haemoglobin content in fish muscle, we found significantly more haemoglobin in the loin of fish left in the net for 24 h. This means that the physiological evidence of traumatic injuries and stress take place prior to quality degradation becoming evident, meaning that muscle haemoglobin is not necessarily a functional welfare indicator for the stress level. That said, despite good quality not always equalling good welfare, poor quality, as measured by haemoglobin content, is evidence of poor welfare.

Given the sheer complexity of interpreting fish welfare, it is clear that relying upon a single parameter, such as lactate, would not provide a complete understanding of the fish’s physiological state. Were to focus purely on lactate, we might reasonably assume that the fish were recovering from the initial escape response while in the net as their behaviour indicated a rested state, i.e. they rarely moved, even when not fully entangled. However, this assumption was not supported by the other physiological stress parameters. When we looked at other indicators such as cortisol, glucose, and haemoglobin, there was no evidence of recovery as soak time progressed. This further points to the complexity of interpreting fish welfare, as different stress markers can tell us different things regarding their physiological condition. Relying on lactate levels alone would give us an incomplete picture. Instead, our findings emphasise the need for a broader approach, using a range of physiological parameters to understand welfare better. This approach is crucial for capturing the full scope of stress and recovery, helping us assess the actual condition of the fish.

### Animal welfare implications

The findings of this study have important implications for animal welfare in fisheries. The increased mortality and physiological stress observed in fish subjected to longer soak times in gillnets show the negative impact that current fishing practices can have on fish welfare. However, adjusting soak times and improving handling practices may not be straightforward because they can affect the efficiency of the gear. Our study demonstrates that animal welfare is compromised during gillnet fishing, and future work on fishing gear should focus on improving animal welfare while maintaining gear efficiency.

## Conclusion

This study demonstrates that capture by gillnets causes significant physiological stress and that longer soak time causes higher mortality and reduced quality, as measured by haemoglobin in the loin. Capture by gillnets appeared to result in a continuous stress response in Atlantic cod. Blood and serum cortisol, magnesium, creatinine, iron and blood glucose levels were useful biomarkers identifying this particular stress response. However, whole blood lactate identified a struggle at the beginning of capture, not the continuing physiological response. Additionally, physiological evidence of stress and injury can occur before visible quality degradation becomes apparent. This suggests that acceptable meat quality is not necessarily equate to good welfare; however, poor quality strongly indicates poor welfare. It is important to note that these findings are specific to Atlantic cod under controlled tank conditions and should not be generalised to all species or fishing practices. Further research is needed to explore the relationship between welfare and quality in other species and fishing contexts.
